# Flow cytometric analysis of equine bronchoalveolar lavage fluid cells in horses with and without severe equine asthma

**DOI:** 10.1177/03009858211042588

**Published:** 2021-09-14

**Authors:** Heng Kang, Dorothee Bienzle, Gary Kwok Cheong Lee, Érica Piché, Laurent Viel, Solomon Olawole Odemuyiwa, Janet Beeler-Marfisi

**Affiliations:** 1 University of Guelph, Guelph, Ontario, Canada; * Current address: Department of Veterinary Pathobiology, College of Veterinary Medicine, University of Missouri, Columbia, MO, USA.

**Keywords:** antibody titration, BALF, flow cytometry, heaves, immunocytochemistry, immunophenotype, macrophages, scavenger receptor, recurrent airway obstruction

## Abstract

Severe equine asthma (SEA) is a common, debilitating lower airway inflammatory disorder of older horses. Alveolar macrophages (AMs) survey inhaled particulates from barn sources causing them to switch from an anti-inflammatory to a proinflammatory phenotype, resulting in neutrophil recruitment to the lung. This proinflammatory switch may contribute to the development and prolongation of SEA. Validated antibodies to identify the cells involved in the pathogenesis of SEA are lacking. In this study, monoclonal antibodies against CD90, CD163, and CD206 were tested for reactivity with equine leukocytes by immunocytochemistry and flow cytometry. A multi-color flow cytometric assay was developed to identify leukocytes in equine bronchoalveolar lavage fluid (BALF). Four control and 4 SEA-susceptible horses had BALF collected before and after a 48-hour moldy hay challenge. Antibodies against CD90 uniquely labeled equine neutrophils, and antibodies against CD163 and CD206 identified equine macrophages. Postchallenge AM surface expression of CD163 increased in both groups of horses, but the increase was statistically significant in only the SEA-susceptible group (*P* = .02). The surface expression of CD206 on AMs increased significantly in the SEA-susceptible group (*P* = .03) but was unchanged in the control group (*P* = .5). Increased expression of CD163 and CD206 during exacerbation of SEA suggested an association between AM phenotype and lung inflammation. However, functions of AMs in the pathogenesis of SEA remain to be elucidated.

Severe equine asthma (SEA) is a chronic noninfectious inflammatory disorder of the distal airway of mature horses.^12^ By a conservative estimate, it has a prevalence of 14% in the northern hemisphere.^16,21,31^ This disease is sufficiently similar to allergic and nonallergic forms of human asthma to justify changing the disease name from recurrent airway obstruction to “severe equine asthma.”^6,12^ Exposure to respirable irritants, including organic and inorganic dusts in the stable, causes exacerbation of SEA.^5,23,39^ Horses affected by SEA have a marked accumulation of neutrophils and mucus in the distal airways and bronchoconstriction leading to airway obstruction. Persistent exacerbation results in long-term airway inflammation leading to airway remodeling and ultrastructural changes in the bronchoalveolar tree.^10,15,26,27,30^ Although clinical signs are improved by reducing barn- and feed-related dusts, and drug therapy, the disease is progressive and periods of exacerbation result in reduced quality of life due to severe respiratory impairment.^3,38,42^ In experimental models, SEA exacerbation can be induced in susceptible horses through exposure to dusty hay or a mixture organic and inorganic particulates and lipopolysaccharide, which represent the asthma triggering components of hay dust.^5,26^ Because the inflammation associated with SEA is diffuse, a single bronchoalveolar lavage fluid (BALF) sample is representative of the process affecting the distal airways.^33^ Cytologically, SEA is defined by counting >25% neutrophils, and variable proportions of lymphocytes and alveolar macrophages (AMs) in the BALF differential cell count.^12^

The immunological basis of SEA has not been fully elucidated. As critical modulators of the inflammatory response, AMs constantly assess inhaled environmental materials. If AMs encounter a specific irritant or infectious agent, they switch to a proinflammatory immunophenotype, but once the agent has been cleared, AMs switch to an anti-inflammatory immunophenotype that restores lung homeostasis.^14,51^ In human asthma, dysregulation of this phenotypic switch can occur secondary to repeated exposure to allergens or pathogens.^14^ This dysregulation results in a proinflammatory AM phenotype that can lead to aberrantly protracted inflammation and worsening of clinical disease.^14^ In horses in exacerbation of SEA, increased T regulatory cells were detected using flow cytometry and increased gene expression of IL-10 was identified in AMs, suggesting that at some point in disease exacerbation, an anti-inflammatory process predominates.^19,50^ However, the connection between the activation status of AMs and asthma has not been fully defined in horses.

Flow cytometry is an effective tool to analyze and quantify multiple characteristics of individual cells in a fluid suspension, including the activation status of AMs.^40^ Macrophages are highly autofluorescent cells because of their flavin and lipid content.^2,37^ This property permits their separation from lymphocytes by flow cytometry;^47^ however, through spectral overlap, the spectrum emitted by macrophage autofluorescence commonly interferes with the detection of fluorescently-conjugated antibodies that label macrophage surface markers.^37^ This issue can be overcome by applying quenching agents,^20^ but such additives are better avoided because of potential signal loss from some fluorophores. Flow cytometry has been applied to immunophenotyping of equine lymphocytes, and peritoneal and alveolar macrophages.^13,40^ But unlike the variety of antibodies available against human and murine cellular antigens,^46^ specific antibodies for equine cellular antigens are very limited, and antibodies developed for other species require validation before use.^24,48^ Additionally, studies using flow cytometry to investigate the distribution of cells within BALF and the phenotype of equine AMs from horses in exacerbation of SEA are rare and incomplete.^19,25^

We hypothesized that in SEA-susceptible horses, AMs undergo an immunophenotypic change between remission and exacerbation states, which can be detected using flow cytometry. The objectives of this study were to (1) validate specific antibodies against equine leukocytes using immunocytochemistry; (2) develop a flow cytometry panel to investigate equine BALF cellular distributions; (3) determine the activation status of AMs by surface expression of CD163 and CD206—typical anti-inflammatory markers in humans;^1,8^ and (4) analyze expression of CD163 and CD206 on AMs in the context of SEA. For the latter, a flow cytometry panel was applied to BALF cells collected from both healthy and SEA-susceptible horses before and after exposure to moldy hay.

## Materials and Methods

### Immunocytochemistry

Immunocytochemistry was performed on charged slide cytocentrifuge (41 × *g* for 6 minutes) preparations of BALF leukocytes from a horse in exacerbation of SEA. Primary antibodies are listed in Table 1. Cytocentrifuge preparations were fixed with cold acetone, incubated with primary antibodies for 30 minutes, washed, and incubated with horseradish peroxidase–labeled secondary anti-IgG antibody (EnVision HRP, Dako Cytomation) for 30 minutes. Preparations were washed and counterstained with hematoxylin, and bound antibodies were detected with NovaRED chromogen (Vector Laboratories). Stained samples were examined by light microscopy to determine antibody specificity.

**Table 1.** Optimized concentrations of antibody for flow cytometric evaluation of cells in equine bronchoalveolar lavage fluid.

Antibody	Clone	Isotype	Concentration	Source
Mouse anti-human CD163	Ber-Mac3	IgG1	0.005 μg/μL	Novus Biologicals, Oakville, Ontario, Canada
Mouse anti-canine CD90	DH24A	IgM	0.005 μg/μL	Washington State University, Pullman, WA
Rat anti-mouse IgG1	M1-14D12	IgG	0.001 μg/μL	Thermo Fisher Scientific, Mississauga, Ontario, Canada
Rat anti-mouse IgM	RMM-1	IgG2a	0.2 μg/μL	BioLegend, San Diego, CA
Mouse anti-human CD206	3.29B1	IgG1	1.0 μg/μL	Beckman Coulter, Mississauga, Ontario, Canada
Mouse anti-horse CD5	CVS5	IgG1	0.1 μg/μL	Bio-Rad, Mississauga, Ontario, Canada
Mouse anti-horse PanB	CVS36	IgG1	1.0 μg/μL	Bio-Rad, Mississauga, Ontario, Canada

### Antibodies for Flow Cytometry

The unconjugated primary antibodies used in this study were mouse anti-canine CD90 and mouse anti-human CD163 (Table 1). Their corresponding secondary antibodies were Brilliant Violet (BV) 510 conjugated rat anti-mouse IgM (clone RMM-1, BioLegend) and phycoerythrin-cyanine7 (PE-Cy7) conjugated rat anti-mouse IgG1 (clone M1-14D12, Thermo Fisher Scientific). The fluorescent primary antibodies used in this study were phycoerythrin (PE) conjugated mouse anti-human CD206 (clone 3.29B1, Beckman Coulter), fluorescein isothiocyanate (FITC) conjugated mouse anti-horse CD5 (clone CVS5, Bio-Rad), and PE conjugated mouse anti-horse PanB cells (clone CVS36, Bio-Rad).

### Antibody Titrations

Antibody titrations were performed using peripheral blood mononuclear cells (PBMCs) isolated from EDTA-anticoagulated whole blood of healthy Standardbred racehorses using SepMate tubes (StemCell Technologies) containing a density gradient (Lymphoprep, StemCell Technologies). A serial dilution of each antibody was prepared, beginning with the manufacturer’s recommended dilution and diluted at 1:5 in flow buffer (phosphate-buffered saline containing 2% horse serum, 10 mM EDTA, and 0.2% sodium azide) in 5 consecutive dilutions. One million PBMCs were labeled for each dilution of antibody. The highest staining index (SI) indicated the optimal antibody concentration. This value was determined using the following equation that incorporates median fluorescence intensity of manually gated positive and negative events:


$$SI=\;\frac{MFI\;of\;positive\;events\;-\;MFI\;of\;negative\;events}{\left(84\text{\% }MFI\;of\;negative\;events\;-\;MFI\;of\;negative\;events\right)\;/\;0.995}$$

### Horses and Sampling

The 8 horses used for the flow cytometry experiments were selected from a research herd composed of horses with normal respiratory function, and SEA-susceptible horses.^45^ These horses are fed from large hay bale feeders, and had routine vaccinations and anthelmintic treatments. All procedures were approved by the University of Guelph Animal Care Committee (Animal Use Protocols #4185 and #3816) and followed guidelines of the Canadian Council on Animal Care.

Prior to moldy hay challenge, a complete physical and respiratory tract examination indicated that both control (*n* = 4) and SEA-susceptible (*n* = 4) horses were clinically normal. Bronchoscopic examination confirmed a lack of airway edema or inflammation, and on pulmonary function testing, transpulmonary pressure was ≤10 cm H_2_O in both groups.^5^ Examination of BALF leukocytes by light microscopy confirmed a neutrophil proportion ≤10% in both groups—an expected finding in control horses and an indicator of remission status in SEA-susceptible horses.^5^ Horses were then individually housed in an air conditioned isolation stall and exposed to moldy hay for 48 hours, followed by another complete physical and respiratory tract examination and pulmonary function test, as described previously.^5^ The latter evaluation was used in conjunction with results from BALF leukocyte enumeration by light microscopy to confirm that SEA-susceptible horses were in exacerbation of the disease while control horses were not.

Prior to and following challenge, BALF samples were collected from each horse and processed within 30 minutes of collection. Briefly, BALF samples were centrifuged at 400 × *g* for 10 minutes at 4 °C, with the brake off. Supernatant was decanted and the cell pellet was washed 3 times using chilled flow buffer. The cell pellet was resuspended in 1 mL flow buffer and counted using a MOXI Z mini automated cell counter (Orflo) following the manufacturer’s guidelines.

### Manual Differential Cell Count

A 400-cell manual differential count was performed on each cytocentrifuge preparation.^7^ Horses in exacerbation of SEA were defined as having neutrophil proportions >25%.^12^

### Flow Cytometry

Individual aliquots of 10^6^ cells were distributed into 1.5 mL microcentrifuge tubes and kept on ice for immunolabeling. Each aliquot was incubated with a viability dye (Zombie NIR, BioLegend) on ice for 15 minutes. Cells were washed with 200 µL flow buffer at 400 × *g* for 3 minutes at 4 °C, and resuspended in flow buffer. Ten microliters of pretitrated antibodies were then added to each cell suspension in the order of unconjugated primary antibodies, secondary antibodies, and conjugated primary antibodies (Table 1). Each addition was followed by 15 minutes of incubation on ice and a wash step. After the final wash, cell pellets were resuspended in 1 mL flow buffer and transferred to flow cytometry tubes for analysis using a FACSCanto II Flow Cytometer (Becton Dickinson and Company) using the following strategy.

During data acquisition, immunostained equine BALF cells were analyzed using FACSDiva software (Becton Dickinson and Company). The forward scatter (FSC) used a linear scale, and the side scatter (SSC) used a logarithmic scale. Scales were adjusted to include all cell populations without excluding any events. The photomultiplier tube detector voltage was adjusted using unstained cells so that they were within 10^0^ to 10^1^ fluorescence units in each channel, and the flow cytometer was set to collect a minimum of 20 000 events from each aliquot of cells. For multi-parameter acquisition, where florescence values were composed of more than one color, a compensation matrix was set using single-color stained cells to minimize the negative interference of spectral overlap. We applied a fluorescence minus one strategy to establish each gate; namely, we labeled cells with all fluorophores except the one of interest. For example, when PE-CY7, PE, and BV510 were used together, the compensation for PE-CY7 was adjusted using samples positively stained with PE and BV510.

The highly autofluorescent nature of AMs necessitated fluorescence minus one control analyses, which determined a gating strategy for the analysis of CD163 expression on AMs (Suppl. Fig. S1). The CD90-positive and CD206-positive cells (autofluorescence plus fluorescence of the fluorochrome) were clearly separated from unstained control cells (autofluorescence alone); thus, no fluorescence minus one control analysis was required.

### Data Analyses

Post-acquisition flow cytometric data were analyzed using FlowJo (Becton Dickinson and Company) software. Median fluorescence intensity was assumed to be proportional to the concentration of cell surface proteins. Two-tailed paired *t*-tests were performed to compare pre- and postchallenge conditions. Spearman’s rank correlation was used to determine the correlations between flow cytometric and manual neutrophil and lymphocyte counts. All statistical analyses were performed using GraphPad Prism (GraphPad Software Inc). Statistical significance was set at *P* ≤ .05.

## Results

### Antibody Validation and Development of Flow Cytometry Methods

**Figures 1–4.**
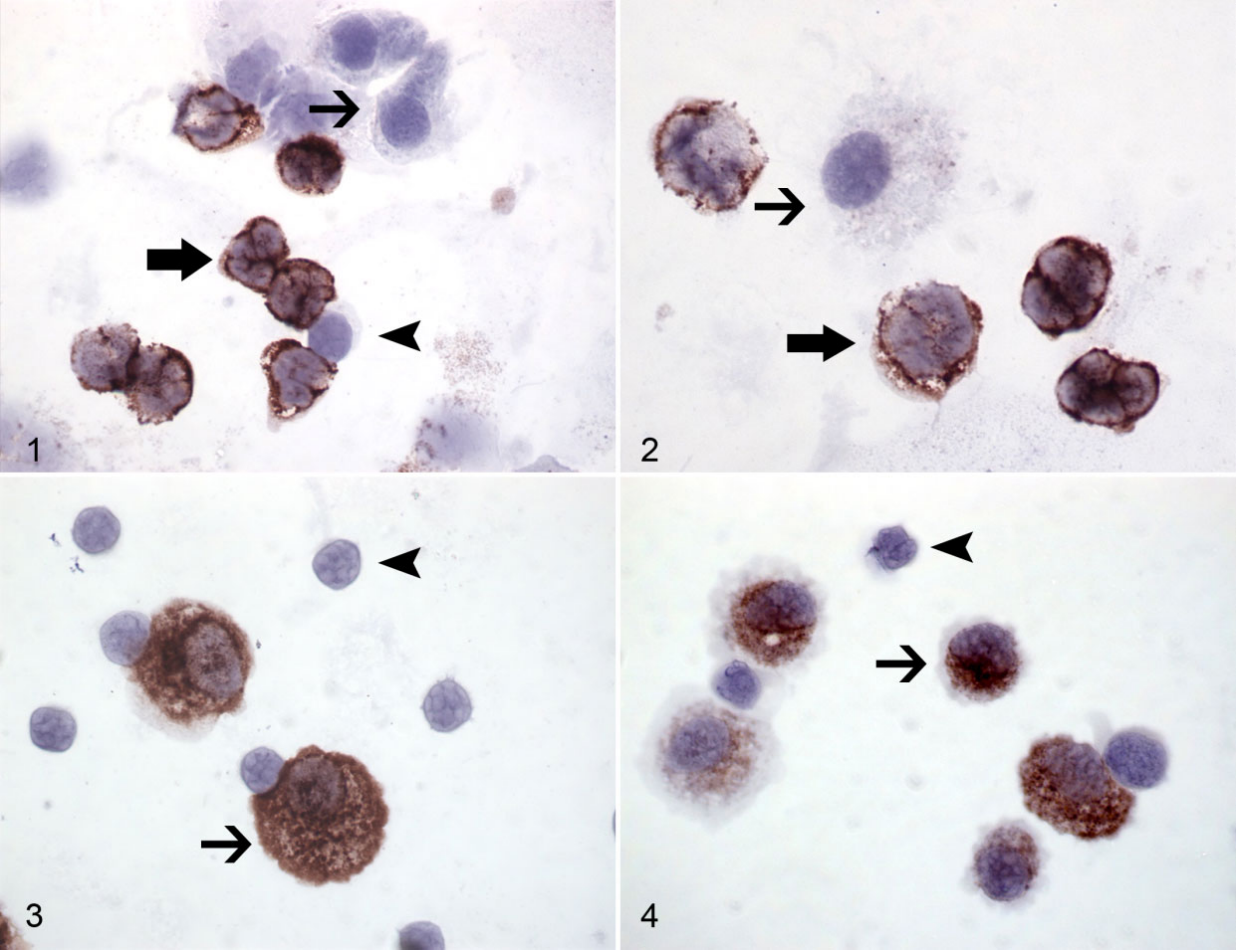
Severe equine asthma, bronchoalveolar lavage cells, horse. Immunocytochemistry for validation of mouse anti-canine CD90 (Figs. 1, 2), mouse anti-human CD163 (Fig. 3) and mouse anti-human CD206 (Fig. 4) antibodies. Antibody to CD90 (Figs. 1, 2) labels neutrophils (thick arrows) but neither lymphocytes (arrowhead) nor alveolar macrophages (thin arrow). Antibodies to CD163 (Fig. 3) and CD206 (Fig. 4) label alveolar macrophages (thin arrows) but not lymphocytes (arrowheads).

Using immunocytochemistry, the anti-CD90 antibody specifically labeled equine neutrophils and not mononuclear cells (Figs. 1, 2). Neutrophils were identified by their segmented nucleus. Anti-CD163 and anti-CD206 antibodies labeled equine alveolar macrophages but neither lymphocytes nor neutrophils (Figs. 3, 4).

Labeling index calculations identified the concentration for each antibody to achieve the optimal separation of positive and negative events (Table 1).

### Flow Cytometric Identification of BALF Cells

**Figure 5.**
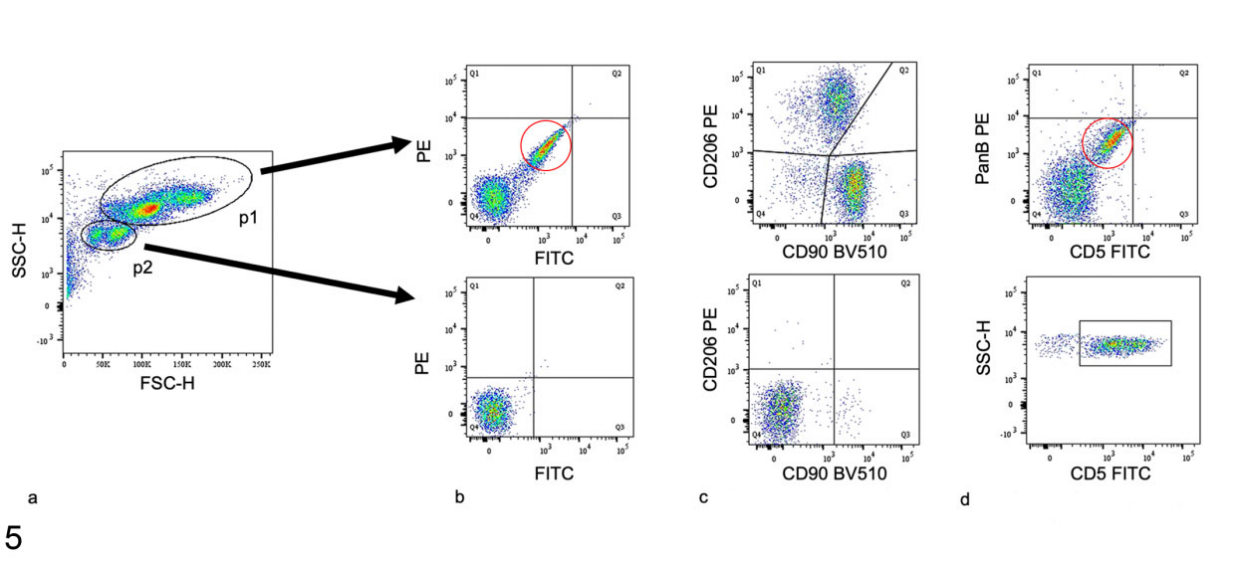
Flow cytometric analysis of equine bronchoalveolar lavage fluid (BALF) leukocytes. (a) Indicates that cells from p1 have higher forward scatter (FSC) and side scatter (SSC) than cells from p2, indicating they are larger and more internally complex. (b) Unstained control sample showing that many (red circle) but not all cells from p1 (upper panel) have autofluorescence in the phycoerythrin (PE) and fluorescein isothiocyanate (FITC) channels. However, cells from p2 (lower panel) are not autofluorescent. (c) Using anti-CD90 (neutrophil marker) and anti-CD206 (macrophage marker) antibodies, cells from p1 (upper panel) but not p2 (lower panel) are identified as neutrophils (Q3) and macrophages (Q1), respectively. (d) Using antibodies against CD5 (lymphocyte marker) and PanB cells, cells in p1 (upper panel) are double-negative, and cells in p2 (lower panel) are identified as lymphocytes; the red circle indicates autofluorescent cells.

Characteristic of equine BALF cells, only 2 populations of cells were identified based on their FSC and SSC properties. Cells from region of interest p1 had moderate FSC and high SSC, and cells from p2 had low FSC and moderate SSC (Fig. 5a). Additional analyses of unstained cells demonstrated that the majority from p1 were autofluorescent in the FITC and PE channels while those from p2 were not (Fig. 5b). CD206^+^ cells (ie, macrophages) and CD90^+^ cells (ie, neutrophils) predominated in p1, while cells from p2 were CD206^-^ and CD90^−^ (Fig. 5c). The majority of cells from p2 were CD5^+^ (ie, lymphocytes; Fig. 5d). No CD5^+^ cells were observed in p1. Therefore, p1 contained highly autofluorescent macrophages and neutrophils, and p2 contained lymphocytes.

### Flow Cytometric and Manual Enumeration of BALF Neutrophils and Lymphocytes

**Figure 6.**
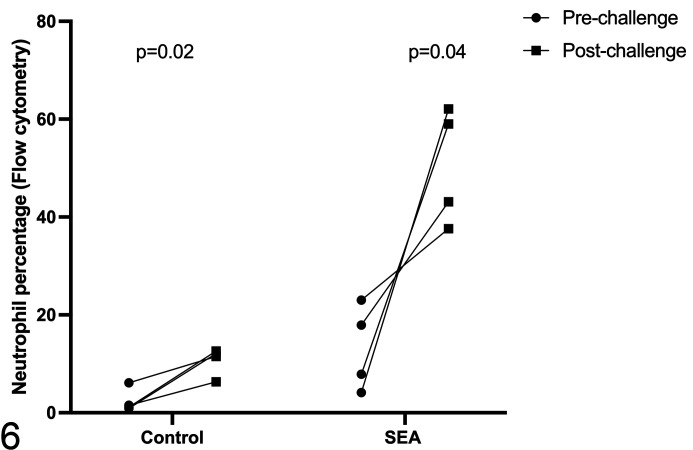
Bronchoalveolar lavage fluid was sampled from control horses and from horses with severe equine asthma (SEA), before and after challenge of the horses with moldy hay. The percentages of neutrophils in bronchoalveolar lavage fluid were higher after moldy hay challenge than prechallenge in both the control group (*n* = 4, *P* = .02) and the SEA group (*n* = 4, *P* = .04).

**Figures 7–8.**
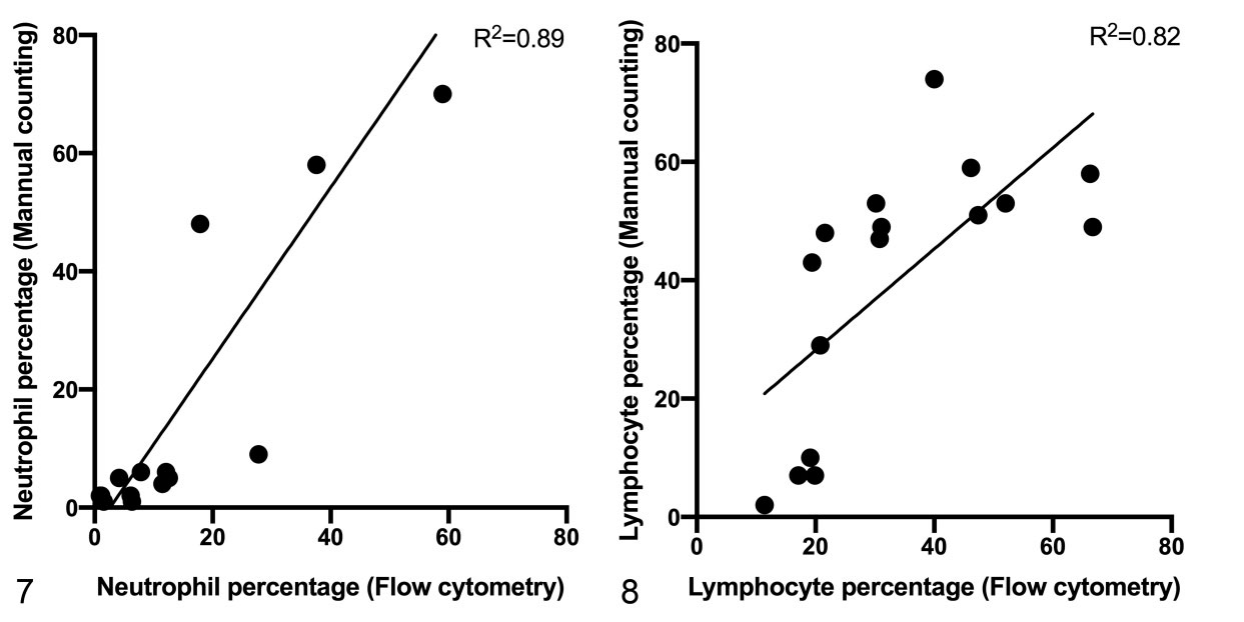
Correlation between flow cytometric enumeration and manual differential cell count to determine the percentage of neutrophils and lymphocytes in bronchoalveolar lavage fluid. Flow cytometric and manual neutrophil and lymphocyte counts are strongly correlated (*n* = 16 BALF samples).

Before and after moldy hay challenge, flow cytometry was used to analyze the percentage of CD90^+^ cells (neutrophils) and CD5^+^ cells (lymphocytes) in both control horses (*n* = 4) and SEA-susceptible horses (*n* = 4). The BALF neutrophil percentage increased significantly after challenge in both groups. Specifically, the mean increase in percentage of neutrophils in control horses was 8.21% (95% confidence interval [CI]: 2.43–13.99; *n* = 4; *P* = .02), whereas that of SEA horses was 36.03% (95% CI: 0.27–71.78; *n* = 4; *P* = .04). The change in neutrophil percentage was significantly different between control and SEA-susceptible horses (*P* = .05). As detected by flow cytometry, the postchallenge neutrophil percentage in BALF of control horses remained less than 20%, whereas that of SEA-susceptible horses increased to 37%, 43%, 59%, and 62% (Fig. 6). There was a strong correlation between flow cytometric counts and manual counts of cytocentrifuge preparations for neutrophils (*R* ^2^ = 0.89) and for lymphocytes (*R* ^2^ = 0.82; Figs. 7, 8).

### Assessment of AM Surface Expression of CD163 and CD206

**Figures 9–10.**
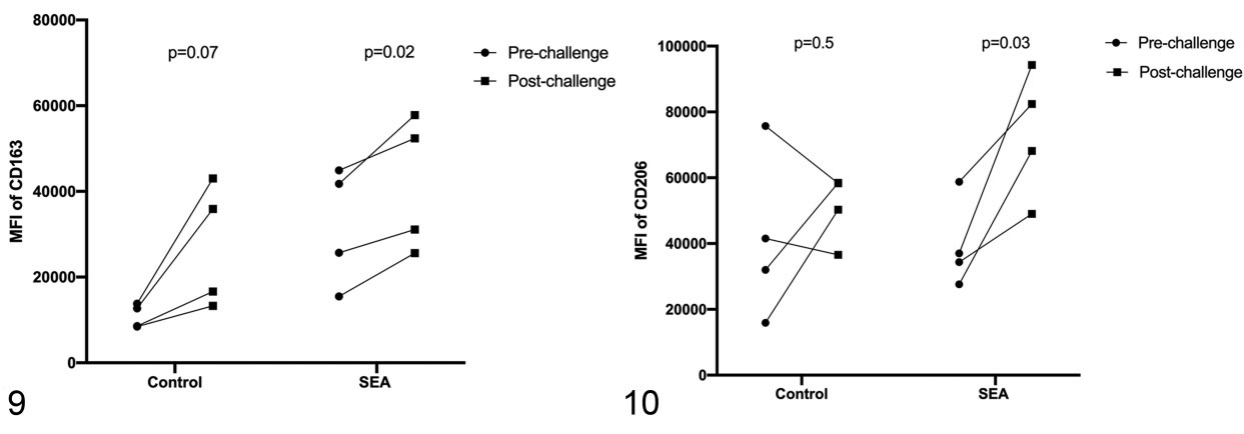
Surface expression of CD163 and CD206 on alveolar macrophages. CD163 expression increased in both groups of horses after undergoing a moldy hay challenge (*n* = 4 in each group), but the increase is significant only in the asthmatic group (Fig. 9). There is a significant increase in CD206 expression in the severe equine asthma-affected (SEA) group (*n* = 4) after moldy hay challenge, but no significant difference in CD206 expression was detected in the control group (*n* = 4; Fig. 10). MFI = mean fluorescence intensity.

Alveolar macrophages in both groups had increased CD163 median florescence intensity after challenge (control: *P* = .07; SEA-affected: *P* = .02; Fig. 9). Postchallenge median fluorescence intensity of CD206 on AMs was significantly higher than prechallenge in the SEA-susceptible group (*P* = .03) but did not show a significant change in the control group (*P* = .5; Fig. 10).

## Discussion

Monoclonal antibodies are integral to immunophenotyping studies. However, particularly in equine research, validated antibodies are lacking and use of nonvalidated antibodies impedes the quality and consistency of data, and reduces the reproducibility of an experiment.^46^ Immunohistochemistry has been performed on equine uveal tract and equine gastrointestinal tract tissues using anti-CD163 antibodies to identify macrophages.^32,43^ However, the clones of those antibodies were different from the current study. Validation of anti-CD206 antibody has not been previously reported in horses; however, immunohistochemistry using anti-CD206 as a macrophage marker was used in human lung sections.^44^ Immunocytochemistry is a useful strategy for antibody validation.^46^ In the present study, immunocytochemistry results showed the specificity of anti-CD90 for equine BALF neutrophils, and anti-CD163 and CD206 for equine AMs (Figs. 1–4). This is the first report of immunocytochemical validation of these antibodies in horses and served as a basis for the subsequent flow cytometry experiments.

In flow cytometry, failure to optimize antibody concentration causes increased nonspecific binding, decreased binding sensitivity, decreased correlation between fluorescence intensity and concentration of marker expression, and increased experimental expense.^11^ Therefore, antibodies were carefully titrated to optimize the flow cytometry experiment in our study (Table 1).

The present study demonstrated that equine BALF cells can be differentiated using multicolor flow cytometry. The flow cytometry panel separated and quantified 3 distinct cell populations including neutrophils, AMs, and lymphocytes, and identified a solution for the problem of AM autofluorescence (Fig. 5). Other groups addressed AM autofluorescence by adding quenching agents to the sample;^20^ however, quenching reduces fluorescence intensity, which can cause signal loss of some fluorophores. In contrast, the current study took advantage of macrophage and neutrophil autofluorescence in the short wavelength channels to separate them from lymphocytes, which helped resolve the 3 distinct populations.

BALF differential cell counts were evaluated using flow cytometry and light microscopic enumeration of a cytocentrifuge preparation.^20^ A previous conclusion from evaluating samples from humans with both methods was that manual counting underestimated the percentage of lymphocytes.^20^ In contrast, the current study identified a strong correlation but different values between flow cytometric and manual neutrophil and lymphocyte counts. These previously noted differences might reflect the fact that flow cytometry analyzes tens of thousands of events compared to manual evaluation of only 400 cells, or that cellular distributions on cytologic preparations may be uneven. Therefore, flow cytometry might provide a more accurate and precise counting of BALF cells.

This investigation identified increased surface expression of CD163 by AMs during the development of severe airway inflammation, suggesting a previously unidentified role for macrophages in the pathogenesis of SEA.^25,50^ In humans, the production of soluble CD163 by monocytes was upregulated in sepsis;^35^ however, the association between CD163 expression on AMs and asthma has not been investigated. In the present study, AM expression of CD163 was upregulated after challenge in both groups. In human monocytes, CD163 expression was upregulated through TLR2, TLR4, and TLR5 activation.^49^ Moldy hay contains TLR ligands such as lipoprotein and lipopolysaccharide derived from bacteria and fungi,^5^ which might have contributed to increased CD163 expression in our study. In humans and mice, CD163 was a marker of anti-inflammatory macrophages.^1,9^ However, the expression of CD163 is regulated by various cytokines at different stages of inflammation.^9^ CD163 expression was induced by anti-inflammatory cytokines such as IL-10, but blockade of IL-10 by IFN-γ led to downregulation of CD163 expression.^9,49^ Horses with SEA had significantly decreased IFN-γ gene expression and higher IL-10 mRNA expression than control horses.^4,28,29^ Additionally, a significant increase in Tregs in BALF and increased gene expression of IL-10 was noted in AMs of SEA-susceptible horses in exacerbation of disease.^19,50^ The upregulation of CD163 expression on AMs after 48 hours of moldy hay challenge in our experiment could be explained by increased IL-10 production by Tregs or AMs, but cytokine expression by AMs has not been fully evaluated in SEA-affected horses.^41^ In addition to flow cytometric evaluation of cell markers, future studies should assess cytokine production to more completely define the individual immune response and to refine our understanding of the different phenotypes in equine asthma.

CD206 is a marker of anti-inflammatory activated macrophages in humans.^1,36^ Gene expression of CD206 by AMs in both control and SEA horses was increased after natural hay challenge.^50^ In contrast, the current study identified increased postchallenge expression of CD206 only within the SEA group, which might indicate that the alveolar milieu in the acute stage of SEA exacerbation drives AMs to a different phenotype than would occur in bacterial infection. This is similar to human asthma where the anti-inflammatory phenotype is permissive of disease progression.^18,34^ This finding needs to be corroborated by additional studies before confidently stating that a similar process is at work in asthmatic horses, but appears to run counter to our initial assumption that pro-inflammatory AMs would predominate.^12,43^

Alveolar macrophages are sensitive to pathogens yet are resistant to activation by innocuous antigens.^22^ In humans and rodents, increases in anti-inflammatory AMs was a feature of several inflammatory lung diseases.^14,22^ During antigen exposure, AMs generally act to suppress inflammation and restore homeostasis, which may explain why AMs in the current study adopted an anti-inflammatory phenotype.^14^ Important features of anti-inflammatory macrophages include increased expression of scavenger receptors and increased phagocytic activity.^17^ Moldy hay contains organic and inorganic particulates and antigens; therefore, detection of increased expression of CD163, the hemoglobin-haptoglobin receptor, and CD206, the mannose receptor, could indicate increased phagocytic function of AMs. Additionally, signal transduction through the mannose receptor prevented AMs from initiating a proinflammatory response against invading microorganisms,^22^ which may also explain why AMs developed an anti-inflammatory phenotype in SEA exacerbation in the current study.

The relatively wide CIs in both control and SEA-affected horses indicate that a larger sample size would be required to fully probe the relationships assessed. A larger sample size would have allowed exclusion of horses with neutrophil percentages above 5%,^12^ or horses could have been maintained on a low dust feed until they had no evidence of lower airway inflammation. Nevertheless, this does not impact the interpretation of a fundamental change in AM phenotype during the exacerbation of SEA.

The current study developed a flow cytometry strategy to determine BALF cellular constituents in an equine asthma model. The validated antibodies and flow cytometry protocol presented here accurately enumerated cellular percentages, and analyzed the expression of cell surface markers. This protocol was applied to non-asthmatic horses, and asthmatic horses in remission and exacerbation making it an effective tool to elucidate the role of AMs in the pathogenesis of SEA.

## Supplemental Material

Supplemental Material, sj-pdf-1-vet-10.1177_03009858211042588 - Flow cytometric analysis of equine bronchoalveolar lavage fluid cells in horses with and without severe equine asthma Click here for additional data file. Supplemental Material, sj-pdf-1-vet-10.1177_03009858211042588 for Flow cytometric analysis of equine bronchoalveolar lavage fluid cells in horses with and without severe equine asthma by Heng Kang, Dorothee Bienzle, Gary Kwok Cheong Lee, Érica Piché, Laurent Viel, Solomon Olawole Odemuyiwa and Janet Beeler-Marfisi in Veterinary Pathology
