# Supplementary material for: Flow cytometric analysis of equine bronchoalveolar lavage fluid cells in horses with and without severe equine asthma
Source: Vet Pathol. 2021 Sep 14;59(1):91–9. doi: 10.1177/03009858211042588 (PMC8679174; doi:10.1177/03009858211042588)
Supplement: Supplemental Material, sj-pdf-1-vet-10.1177_03009858211042588 - Flow cytometric analysis of equine bronchoalveolar lavage fluid cells in horses with and without severe equine asthma [file sj-pdf-1-vet-10.1177_03009858211042588.pdf]

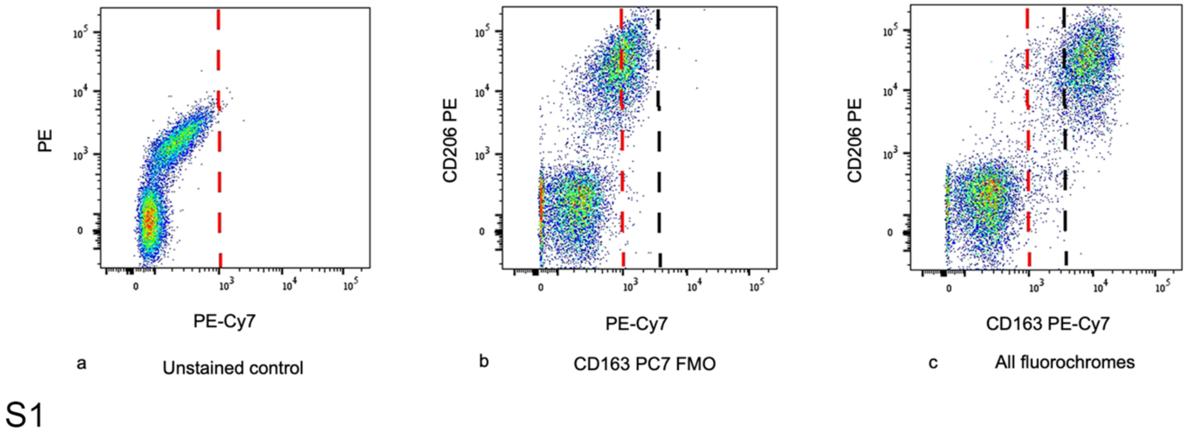

Supplemental Figure 1. Fluorescence minus one controls used to determine an optimal gating strategy for bronchoalveolar lavage fluid (BALF) leukocytes. **a**: unstained control. **b**: CD163 fluorescence minus one control, i.e., BALF cells immunolabeled with every antibody except anti-CD163 phycoerythrin-cyanine 7 (PE-CY7). **c**: fully stained cells. The differences between the unstained boundary (red dashed line) and fluorescence minus one boundary (black dashed line) shows the fluorescence spread in the PE-CY7 channel.
